# Sense of Coherence Mediates the Relationship Between Cognitive Reserve and Cognition in Middle-Aged Adults

**DOI:** 10.3389/fpsyg.2022.835415

**Published:** 2022-03-28

**Authors:** Gabriele Cattaneo, Javier Solana-Sánchez, Kilian Abellaneda-Pérez, Cristina Portellano-Ortiz, Selma Delgado-Gallén, Vanessa Alviarez Schulze, Catherine Pachón-García, H. Zetterberg, Jose Maria Tormos, Alvaro Pascual-Leone, David Bartrés-Faz

**Affiliations:** ^1^Institut Guttmann, Institut Universitari de Neurorehabilitació adscrit a la UAB, Barcelona, Spain; ^2^Departament de Medicina, Universitat Autònoma de Barcelona, Bellaterra, Spain; ^3^Fundació Institut d’Investigació en Ciències de la Salut Germans Trias i Pujol, Barcelona, Spain; ^4^Departament de Medicina, Facultat de Medicina i Ciències de la Salut i Institut de Neurociències, Universitat de Barcelona, Barcelona, Spain; ^5^Institut d’Investigacions Biomèdiques August Pi i Sunyer, Barcelona, Spain; ^6^Departamento de Ciencias del Comportamiento, Escuela de Psicologéa, Universidad Metropolitana, Caracas, Venezuela; ^7^Department of Psychiatry and Neurochemistry, Institute of Neuroscience and Physiology, The Sahlgrenska Academy at the University of Gothenburg, Mölndal, Sweden; ^8^Clinical Neurochemistry Laboratory, Sahlgrenska University Hospital, Mölndal, Sweden; ^9^Department of Neurodegenerative Disease, UCL Institute of Neurology, London, United Kingdom; ^10^UK Dementia Research Institute at UCL, London, United Kingdom; ^11^Hong Kong Center for Neurodegenerative Diseases, Shatin, Hong Kong SAR, China; ^12^Hinda and Arthur Marcus Institute for Aging Research and Deanna and Sidney Wolk Center for Memory Health, Hebrew SeniorLife, Boston, MA, United States; ^13^Department of Neurology, Harvard Medical School, Boston, MA, United States

**Keywords:** sense of coherence, purpose in life, cognition, cognitive reserve, brain health

## Abstract

In recent years, supported by new scientific evidence, the conceptualization of cognitive reserve (CR) has been progressively enriched and now encompasses not only cognitive stimulating activities or educational level, but also lifestyle activities, such as leisure physical activity and socialization. In this context, there is increasing interest in understanding the role of psychological factors in brain health and cognitive functioning. In a previous study, we have found that these factors mediated the relationship between CR and self-reported cognitive functioning. In this study, we have confirmed an association between two important constructs included in the psychological wellbeing and salutogenic models, “purpose in life” and “sense of coherence,” CR, as assessed using a questionnaire, and cognitive functioning, as evaluated using a comprehensive neuropsychological battery. Results from 888 middle-aged healthy participants from the Barcelona Brain Health Initiative indicate that both sense of coherence (SoC) and CR were positively associated with verbal memory, reasoning and attention, working memory, and global cognition. Moreover, the relation between CR and cognitive functioning in the different domains is partially mediated by SoC. When we controlled for brain integrity, introducing into the model neurofilament light chain measures, the mediator role of SoC was confirmed for reasoning and attention and global cognition. However, purpose in life was not associated with cognitive functioning. These results reveal the central role of the SoC construct, which mediates the association between classic CR estimates and cognitive functions, potentially representing a modifiable target for interventions that aim to promote brain health.

## Introduction

Recent biomedical research is increasingly focused on understanding why a substantial portion of individuals remain cognitively and functionally normal throughout their lifetime, irrespective of the occurrence of age-related brain changes or the presence of brain pathology (Arenaza-Urquijo and Vemuri, 2018). Parallel to experimental evidence that a large number of factors, both modifiable and non-modifiable, play a role and interact in determining different brain and behavioral trajectories during the lifespan (Di Marco et al., 2014; Livingston et al., 2020), classical theoretical models, such as cognitive reserve (CR), are constantly evolving and expanding.

Among the different CR conceptualizations proposed in the past few decades, we considered a dynamic model of reserve that includes those experiences that explain how successfully a person can cope with age, disease-related brain changes, or achieve better performance on cognitive tasks due to better brain network efficiency (Steffener and Stern, 2012). In this sense, CR is considered as “the ability to optimize or maximize performance through differential recruitment of brain networks, which perhaps reflect the use of alternate cognitive strategies” (Stern, 2002). This ability is associated with both early and late-life socio-behavioral experiences pertaining to a broad variety of domains.

Although original conceptualizations of CR mainly considered the roles of education and occupation as proxy variables, in combination with measures of premorbid intelligence estimations (Stern, 2012), a broad variety of activities through life are now being considered (Stern et al., 2020). In this regard, the role of psychological factors, including personality traits, coping strategies, negative thinking, and attitude toward life, has been investigated recently as important factors in promoting brain health and resilience (Franchow et al., 2013; Ryff, 2014; Bartrés-Faz et al., 2018; Arenaza-Urquijo et al., 2020; Colombo et al., 2020; Marchant et al., 2020).

Similarly, different variables included in the psychological wellbeing construct proposed by Ryff (1995) (e.g., Purpose in life, PiL), as well as dimensions included in the salutogenic model of Antonovsky (1993) (sense of coherence, SoC), have been related with higher levels of mental health *via* stress management and self-regulation mechanisms (Durand-Bush et al., 2015; Mc Gee et al., 2018). These eudaimonic dimensions, encompassing the experience of fulfillment achieved through self-actualization and a goal-directed existence, have been consistently considered as protector factors for biological markers related to the stress pathway, such as cortisol levels (i.e., Pressman et al., 2019) and inflammatory markers (i.e., Ironson et al., 2018), and have been shown to interact and moderate the relation between classical proxies of CR and inflammatory processes (see Ryff, 2014 for a review).

Sense of coherence represents the central factor on the salutogenic model, originally proposed by Antonovsky (1990). This construct is defined as a “global orientation that expresses the extent to which one has a pervasive, enduring though dynamic feeling of confidence that: (1) the stimuli deriving from one’s self internal and external environments in the course of living are structured, predictable, and explicable; (2) the resources are available to one to meet the demands posed by these stimuli; and (3) these demands are challenges, worthy of investment and engagement” (Antonovsky, 1996). Hence, SoC has a cognitive and behavioral-instrumental dimension, being considered the “cognitive” component of the meaning in life theory (Martela and Steger, 2016). This cross-cultural construct represents a general orientation in promoting health by allowing individuals to actively use available internal and environmental resources to cope with adverse events and, ultimately, to boost resilience to them (Antonovsky, 1990).

Epidemiological and clinical studies have shown that SoC is an important health-promoting resource, and it has been related to the quality of life and a broad range of health-related variables, as well as to the prevalence of distinct pathological conditions, including depression, cardiovascular accidents, and mortality (Lundman et al., 2010; Boeckxstaens et al., 2016; Mittelmark et al., 2016; Huang et al., 2017).

Sense of coherence has also been consistently associated with classical proxies of CR, such as education (Giglio et al., 2015) and leisure-time physical activity (Monma et al., 2015), and has been positively associated with both self-reported (Read et al., 2005) and measured cognitive functioning (Boeckxstaens et al., 2016). Interestingly, a longitudinal study in older adults observed a parallel decline in cognition and SoC over time (Lövheim et al., 2013), supporting the role of this construct in cognition and optimal functioning.

Purpose in life, instead, represents a motivational psychological construct relating to the notion of having a sense of future-oriented and meaningful goals in life. It is one of the six factors composing the psychological wellbeing model proposed by Ryff (1995), together with autonomy, personal growth, environmental mastery, positive relationships, and self-acceptance. Higher levels of PiL could moderate the impact of biological risk factors, such as inflammatory markers, and may be associated with a better capacity to face challenging conditions that could affect mental and physical health (see Ryff et al., 2016 for a review). It has been shown that PiL is related to a reduction in the incidence of various health-related conditions, such as stroke, cardiovascular accidents, disability, and other causes of mortality (Kim et al., 2013; Cohen et al., 2016; Kim and Park, 2017). In the context of cognition and dementia, PiL has been associated with better cognitive function in healthy adults, reduced incidence of mild cognitive impairment (MCI) and risk of developing Alzheimer’s disease (AD), and crucially with better cognitive functioning in the presence of more AD pathology (Boyle et al., 2010, 2012; Lewis et al., 2016).

The associations between psychological constructs and cognitive outcomes, as well as their consistent relation with classical proxies of CR, suggest their possible role in the relation between CR and cognition. In fact, higher estimates of these constructs could influence classical proxies of reserve (e.g., social aspects), or be influenced by them (e.g., educational level), modifying their relationship with cognitive functioning.

In a previous study (Bartrés-Faz et al., 2018), we already observed that both SoC and PiL mediate the relation between classical proxies of CR and self-perceived cognitive functioning. In this study, we wanted to make a further step by exploring the relationship between these psychological constructs, CR, and objective cognitive performance. This was assessed through a formal neuropsychological evaluation in a large sample of middle-aged and older adults (1) using objective measures of cognitive functioning and (2) exploring different cognitive domains separately.

In line with the results of our previous study, we hypothesized that SoC, seen as a psychological cognitive construct related to resources control and managing in response to environmental demands, would be particularly associated with general cognitive functioning and executive functions, while PiL may show a weaker association with cognitive measures. To achieve the objectives linked to these hypotheses, we employed a “controlling” model that explores the association between proxies of CR and cognition, and the mediator role of two psychological constructs, in light of similar levels of brain status in healthy middle-aged adults.

## Materials and Methods

### Participants

In the framework of the Barcelona Brain Health Initiative (BBHI) (see Cattaneo et al., 2018, 2020 for details), 888 participants (433 women, mean age = 53.35, standard deviation = 7.1, range = 42–67) that underwent in-person assessments participated in the study. Participants were selected if they accomplished a cognitive assessment and completed an online survey on CR (Rami et al., 2011), SoC, and PiL estimates (Díaz et al., 2006; Virués-Ortega et al., 2007). Furthermore, participants did not present with any neurological or psychiatric diagnosis at the time of entering the study.

### Assessment

#### Neuropsychological Assessment

Neuropsychological testing was administered by two expert neuropsychologists (V.A. and C.P.) in a single session of approximately 90 min (see Cattaneo et al., 2018). Tests were administered in a fixed order: direct and inverse digit span (Peña-Casanova et al., 2012), Trail Making Test Parts A and B (Peña-Casanova et al., 2012), Reasoning Matrix (Weschler, 2008), Rey Auditory-Verbal Learning Test (Bowler, 2013), block design test (Weschler, 2008), letter-number sequencing (Peña-Casanova et al., 2012), digit-symbol substitution test and cancelation subtests from Wechsler Adult Intelligence Scale, Fourth Edition (WAIS-IV) (Weschler, 2008), and Corsi block-tapping test (Peña-Casanova et al., 2012).

#### Neurofilament Light Chain Measurement

We collected blood samples using ethylenediaminetetra-acetic acid (EDTA) tubes during the medical assessment of the BBHI, and plasma was aliquoted and stored in a freezer at −80^°^C in a biobank facility following standard procedures usually employed for clinical purposes. Plasma Neurofilament light chain (NfL) concentration, a general marker of neurodegeneration (Teunissen et al., 2022), was measured using the single-molecule array (Simoa) NF-light Advantage Kit on an HD-X instrument as described by the kit manufacturer (Quanterix, Billerica, MA, United States). The limit of quantification was 2.7 pg/ml, and the limit of detection was 0.3 pg/ml. For the quality control (QC) sample with an 11.2 pg/ml concentration, repeatability was 3.6%, and intermediate precision was 5.0%. For a QC sample with a 115 pg/ml concentration, repeatability was 5.3%, and intermediate precision was 6.8%. The measurements were performed at the Clinical Neurochemistry Laboratory at the University of Gothenburg by board-certified laboratory technicians who were blinded to clinical data.

#### Online Surveys

*Cognitive reserve* was estimated using a short questionnaire validated in the Spanish population and previously used in studies with healthy elders and patients with AD (Rami et al., 2011). The questionnaire obtained information about six main proxies of CR entailing attained level of education, occupation, musical formation, language skills, reading activity, and intellectual games. The total score (from 0 to 25) was obtained by directly adding the single response scores. Higher scores in this questionnaire correspond to higher levels of CR (refer to Table 1).

**TABLE 1:** Results of online surveys.

Survey	Mean (SD)
Cognitive reserve (CR)	19.76 (3.62)
Sense of coherence (SOC)	66.92 (12.09)
Purpose in life (PiL)	28.73 (6.05)

*Sense of coherence* was evaluated using the Spanish version of the orientation to life questionnaire (OLQ-13; Lizarbe-Chocarro et al., 2016). This 13-item scale represented an abbreviated version of the original 29-items tool and uses a 7-point Likert-type response scale (range: 0–91). Higher scores in this scale correspond to higher levels of SoC (refer to Table 1).

*Purpose in life* was measured using the PiL subscale of the Spanish version of Ryff’s wellbeing scale (van dierendonck et al., 2008) that with a Likert-type scale ranging from 1 (strongly disagree) to 6 (strongly agree), whereby higher scores correspond to higher levels of PiL (refer to Table 1).

### Statistical Analysis

First, as performed in previous studies of our group (e.g., España-Irla et al., 2021), we transformed raw scores obtained by cognitive tests to *z*-scores, then, to create composite scores of different cognitive domains, we ran an exploratory principal component analysis using Oblimin rotation and fixed the acceptable level of factor loading to 0.30 (Hair et al., 1998). Second, we explored the association between SoC and PiL by running Spearman correlations.

Finally, we ran two multivariate linear regression models, one for SoC and another for PiL, to control for collinearity due to the high correlation between these two measures, using the different composite cognitive scores (see below) as dependent variables and CR, age, gender, and the psychological construct estimation as regressors. Based on the results obtained, when possible, mediation analyses were undertaken using the Preacher and Hayes’ mediation procedure (Preacher and Hayes, 2008). Models were repeated in a subsample (*N* = 756) where NfL data were available. Here, NfL was used as a covariate in the analyses to adjust for neurodegeneration.

All statistical analyses were carried out using the SPSS version 23.0 software (Statistical Package for Social Sciences, Chicago, IL, United States), and mediation analyses were carried out using the PROCESS macro for SPSS (Preacher and Hayes, 2008).

## Results

### Cognitive Composite Score Calculation

Neuropsychological raw data of the 888 participants (refer to Table 2) were transformed into *z*-scores and used in the principal component analysis. Bartlett’s test revealed a significant relationship between the factors (*p* < 0.001), and the Kaiser-Meyer-Olkin (KMO) test confirmed that the data were suitable for factor analysis (KMO = 0.63).

**TABLE 2:** Results of formal neuropsychological testing of participants.

Neuropsychological test	Mean (SD)
Digit span forward	6.14 (1.21)
Digit span dackward	4.92 (1.10)
Letter-number sequencing	5.74 (1.04)
RAVLT immediate recall	51.84 (8.65)
RAVLT delayed recall	11.31 (2.68)
RAVLT recognizing	14.35 (1.20)
WAIS-IV logical matrices	19.97 (3.39)
WAIS-IV block design	46.10 (10.44)
Digit symbol substitution test	77.90 (13.41)
WAIS-IV Cancelation	41.72 (8.38)
TMT A	27.51 (8.69)
TMT B	79.14 (26.34)
TMT B-A	51.60 (24.64)

The principal component analysis resulted in four components, the first included all measures of the Rey Auditory Verbal Learning Test (immediate recall = 0.88, delayed recall = 0.91, and recognition = 0.79), indicating a verbal memory domain. A second reasoning and attentional component comprised the Trail Making Test A (0.77), the block design test (0.74), the digit-symbol substitution test (0.70), the WAIS-IV matrix reasoning (0.65), and the cancelation test (0.53). The third factor reflecting working memory contained digit span backward (0.79), digit span forward (0.78), and the letter-number sequencing tests (0.70). Finally, set-shifting abilities were reflected in a fourth component, with the Trail Making Test Part B (0.97) and the Trail Making Test Part B-A (0.97). Based on the factorial structure obtained, we calculated composite scores of the four domains as the mean of *z*-scores of each neuropsychological test. Moreover, we calculated a global cognition composite as the mean of all the transformed *z*-scores.

### Sense of Coherence and Purpose in Life

Spearman correlations revealed a very strong positive association between SoC and PiL (ρ = 0.68, *p* < 0.001).

### Sense of Coherence, Cognitive Reserve, and Cognition

#### Verbal Memory

The multivariate regression model results were statistically significant (*F* = 45.91, *p* < 0.001) and explained 21% of the variance.

**FIGURE 1:**
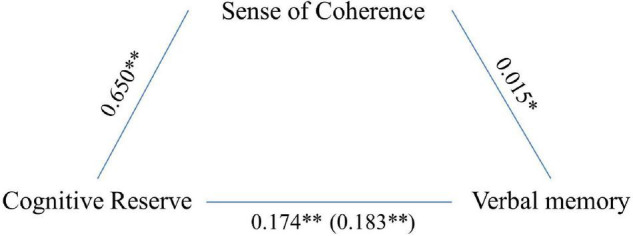
Sense of coherence (SoC) as a partial mediator of the association between CR and verbal memory. Values are *B* coefficients (*p < 0.05; **p < 0.01); values within parentheses represent total relationship.

Analyses revealed that memory was associated with age (*F* = 78.071, *p* < 0.001), gender (*F* = 63.851, *p* < 0.001), CR (*F* = 61.990, *p* < 0.001), and SoC (*F* = 5.110, *p* = 0.024). In addition, bootstrapped mediation analysis revealed that SoC significantly and partially mediated the association between CR and cognitive functioning in this domain (5,000 bootstrap samples, 95% CI 0.002–0.020; see Figure 1). The model explained 19% of the variance without mediators and 20% when SoC was included as a mediator.

#### Reasoning and Attention

In this case, the multivariate regression model results were statistically significant (*F* = 69.50, *p* < 0.001) and explained 28% of the variance.

**FIGURE 2:**
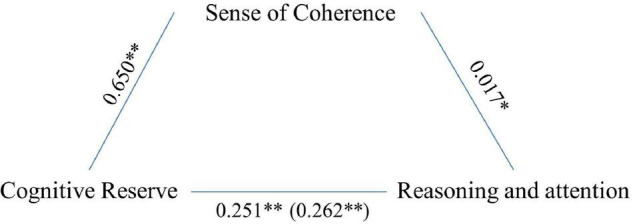
Sense of coherence as mediators of the association between CR and reasoning. Values are *B* coefficients (*p < 0.05; **p < 0.01); values within parentheses represent total relationship.

Reasoning and attention were associated with age (*F* = 259.722, *p* < 0.001), CR (*F* = 82.867, *p* < 0.001), and SoC (*F* = 4.258, *p* = 0.039). Also, in this case, mediation analysis indicated that SoC significantly and partially mediated the association between CR and cognitive performance (5,000 bootstrap samples, 95% CI 0.002–0.024; see Figure 2). The model explained 27% of the variance without mediators and 28% when SoC was introduced as a mediator.

#### Working Memory

Multivariate regression model results were statistically significant (*F* = 15.43, *p* < 0.001) but explained only 8% of the variance.

**FIGURE 3:**
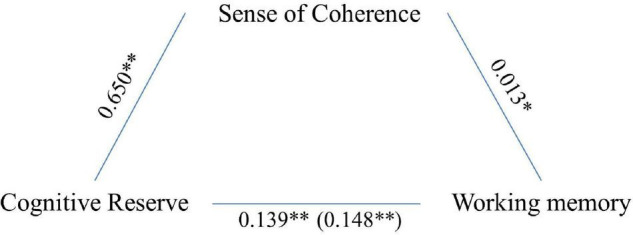
Sense of coherence as a partial mediator of the association between CR and working memory. Values are *B* coefficients (*p < 0.05; **p < 0.01); values within parentheses represent total relationship.

However, working memory results were associated with age (*F* = 12.571, *p* < 0.001), gender (*F* = 11.752, *p* < 0.001), CR (*F* = 44.183, *p* < 0.001), and SoC (*F* = 4.439, *p* = 0.035). Further mediation analysis indicated that SoC significantly and partially mediated the association between CR and cognitive functioning in this domain (5,000 bootstrap samples, 95% CI 0.001–0.018; see Figure 3). The model explained 7% of the variance without mediators and rose to 8% when SoC was included as the mediator.

#### Set Shifting

The whole regression model results were statistically significant (*F* = 35.83, *p* < 0.001) and explained 16% of the variance.

Set shifting results were only associated with age (*F* = 121.658, *p* < 0.001) and CR (*F* = 53.326, *p* < 0.001) but not with SoC (*F* = 0.001, *p* = 0.973).

#### Global Cognition

For global cognition, the multivariate model results were statistically significant (*F* = 83.40, *p* < 0.001) and explained 32% of the variance.

**FIGURE 4:**
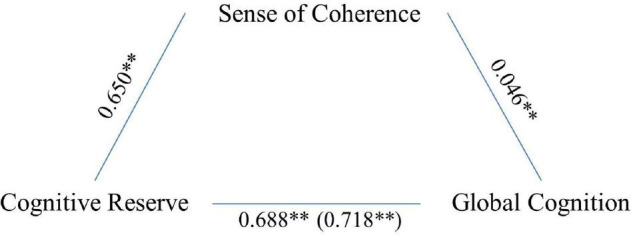
Sense of coherence as a partial mediator of the association between CR and global cognition. Values are *B* coefficients (*p < 0.05; **p < 0.01); values within parentheses represent total relationship.

Global cognition results were associated with age (*F* = 78.071, *p* < 0.001), CR (*F* = 144.702, *p* < 0.001), and SoC (*F* = 6.995, *p* = 0.008). Again, mediation analysis indicated that SoC significantly and partially mediated the association between CR and cognitive performance (5,000 bootstrap samples, 95% CI 0.009–0.056; see Figure 4). The model explained 31% of the variance without mediators and 32% with SoC as a mediator.

#### Neurofilament Light Chain

We repeated the previous analysis in a subsample (*N* = 756), introducing NfL as a covariate to confirm our results that SoC exerted a modulation effect, while accounting for a measure reflecting brain status, as recommended in the operational definitions of CR by Stern and collaborators (Stern et al., 2020). CR was significantly associated with all cognitive domains, while SoC was associated with reasoning and attention (*F* = 4.328, *p* = 0.038) and global cognition (*F* = 5.317, *p* = 0.021) and only partially associated with episodic memory (*F* = 3.045, *p* = 0.081) and working memory (*F* = 2.862, *p* = 0.091). When we ran mediation models for significantly associated domains, we found that SoC partially mediated the relationship between CR and reasoning and attention (5,000 bootstrap samples, 95% CI 0.002–0.028) and global cognition (5,000 bootstrap samples, 95% CI 0.007–0.061).

### Purpose in Life, Cognitive Reserve, and Cognition

Multivariate analysis revealed that PiL was not significantly associated with verbal memory (*F* = 0.326, *p* = 0.568), reasoning and attention (*F* = 0.851, *p* = 0.356), working memory (*F* = 0.037, *p* = 0.847), cognitive flexibility (*F* = 1.373, *p* = 0.242), or global cognition (*F* = 0.003, *p* = 0.957). Results were confirmed when we introduced NfL in the model as a covariate.

## Discussion

In this study, we explored the relationship between two psychological constructs (SoC and PiL), CR, and cognition for different cognitive domains. We used a “controlling” model that explores the association between proxies of CR and cognition and the mediator role of two psychological constructs, in light of similar levels of brain status (Stern et al., 2020). In a first analysis, similar brain status was assumed considering the characteristics of our healthy middle-aged participants, while in a second analysis realized in a subsample, we adjusted for a neurodegeneration marker, namely NfL, to confirm the obtained results.

Present findings indicated that CR was associated with all cognitive domains, while SoC was related to composite scores in verbal memory, reasoning and attention, working memory, and global cognition. Moreover, SoC exerted a mediator role on the relationship between CR and all these cognitive measures. When we corrected for a measure of brain status in a subsample, we confirmed previous results but only for reasoning and attention and global cognition. Instead, PiL did not reveal clear associations with cognitive functioning.

Our findings regarding SoC add further new evidence to our previous study on the relationship between components of meaning in life and self-reported cognitive status, cognitive complaints, and classical proxies of CR (Bartrés-Faz et al., 2018). In particular, they highlight the presence of an association between the psychological construct of SoC and cognitive functioning measured through formal neuropsychological assessments, as well as its mediator role on the relation between CR and cognition. As highlighted in the introduction, SoC is considered a psychological construct involved in resources control and the ability to manage these resources to better respond to environmental demands. Similarly, in its classical formulation, CR estimations were thought to reflect mechanisms that allow them to manage and optimally employ available resources to cope with brain changes and insults (Stern, 2009). Present results revealed that the association between these two constructs with cognitive functioning is not limited to some cognitive domains, but rather it seems to be spread across different cognitive functions. However, when we corrected for brain status, SoC was associated with global cognition and reasoning and attention, suggesting both general and specific effects on cognition of this construct. These findings parallel recent proposals considering that CR relates to better cognitive performance in multiple areas of cognition (Stern et al., 2018; van Loenhoud et al., 2020), and it is not specifically associated with executive functions, as initially proposed (Tucker and Stern, 2011; Roldán-Tapia et al., 2012).

Recently, Stern et al. (2018), using an iterative approach, identified a CR neural network that included brain regions forming parts of main large-scale networks, such as the default mode, the frontoparietal, and the salience networks (van Loenhoud et al., 2020), and was found to be involved in the execution of different cognitive tasks. Interestingly, its expression correlated with IQ, a proxy of CR, and crucially moderated the association between performance in some cognitive domains and other classical brain metrics, such as cortical thickness (Stern et al., 2018).

This evidence of a mediator role of CR on the relationship between brain measures and cognitive performance (see also Steffener et al., 2014) clearly suggested that CR not only acts as a mechanism that allows some individuals to better tolerate brain pathology (Stern, 2002) but also facilitates healthy individuals to perform better in cognitive tasks, possibly through promoting neural efficiency (Stern et al., 2018). To our knowledge, no studies have directly explored the neurobiological substrates of SoC at the level of brain network expressions; the cognitive findings may suggest that SoC may promote cognition through partial overlapping of neural underpins. In this regard, literature on neurobiology of stress suggests that prefrontal brain regions involved in emotional regulation, that, in part, overlap with those included in the salience network (Gupta et al., 2017), could be the neural substrate of SoC (Smith, 2002), in line with the hypothesis that these act on the brain and mental health *via* stress management and self-regulation mechanisms (Mc Gee et al., 2018). The only partial mediation effect of SoC on the relationship between CR and cognitive functioning, and the limited increase in the explained variance when it was included in the model as a mediator, suggested that the overlap between CR and SoC is restricted. In fact, when we adjusted analysis for a general neurodegeneration marker, we only see a mediator role of SoC for reasoning and attention (beyond global cognition), suggesting a specific effect only on some cognitive domains. Therefore, beyond the potential influence of SoC on classical proxies of CR (and vice versa) that could explain its mediation role for specific cognitive domains, its complexity as a psychological construct and its relationship with a broad variety of health-related outcomes suggest that it could act *via* independent protective mechanisms (e.g., stress reduction; Kimhi, 2015).

In contrast to SoC findings, PiL was not found to be associated with cognitive functioning in any of the domains considered. Initially, these results are contradictory to the previous investigations that looked at the relationship between PiL, cognition, and cognitive decline in aging. Results from Boyle et al. (2012, 2010) indicate that individuals with higher estimations of PiL are less prone to cognitive decline, MCI, and dementia, and they present better cognitive functioning in the presence of more brain pathology. In contrast to these previous investigations, including participants affected by MCI or in early or advanced stages of AD, we selected cognitive spared, middle-aged individuals and did not have follow-up data on cognitive changes. These methodological differences may account for the observed discrepancies in results. Hence, albeit speculative at this stage, this more future-oriented and motivational construct can exert a relevant role in promoting cognitive resilience in the presence of brain pathology and less impact, or no detectable relevance on cognitive outcomes, when considering normally performing individuals (however, see Merten et al., 2021, to explore the relationship between psychological wellbeing composite score, including PiL, and cognitive functioning and impairments).

Our present findings advocate for the inclusion of the construct of SoC as one of the factors potentially relevant for the study of CR and its relationship with cognitive functioning. This is in accordance with CR being a continuously evolving, dynamic concept, where the relevance of psychological and psycho-affective variables is accumulating (e.g., Marchant and Howard, 2015; Marchant et al., 2020). At a practical level, the incorporation of this kind of aspects in the study of the effect of CR on cognition may be highly relevant, considering that central concepts (such as the identification of intrinsic values that guide behavior, in the case of SoC) are usually amenable to psychological interventions (e.g., Hayes, 2016), therefore opening new avenues to promote reserve.

## Data Availability Statement

The raw data supporting the conclusions of this article will be made available by the authors, without undue reservation.

## Ethics Statement

The studies involving human participants were reviewed and approved by the Fundació Unio Catalana Hospitals (code CEIC 18/07). The patients/participants provided their written informed consent to participate in this study.

## Author Contributions

AP-L, DB-F, and JT participated in the initial conception of the design of the Barcelona Brain Health Initiative. GC and JS-S made substantial contributions to the actual design and implantation protocol. GC, JS-S, VA, and CP-G participated actively in the data collection and analysis. HZ supervised the blood biomarker measurements. KA-P, CP-O, SD-G, JT, DB-F, and AP-L contributed to the interpretation of the results. GC drafted the article. All authors made critical revisions, introducing important intellectual content, and final approval of the submitted version.

## Author Disclaimer

The content is solely the responsibility of the authors and does not necessarily represent the official views of the National Institutes of Health.

## Conflict of Interest

The authors declare that the research was conducted in the absence of any commercial or financial relationships that could be construed as a potential conflict of interest.

## Publisher’s Note

All claims expressed in this article are solely those of the authors and do not necessarily represent those of their affiliated organizations, or those of the publisher, the editors and the reviewers. Any product that may be evaluated in this article, or claim that may be made by its manufacturer, is not guaranteed or endorsed by the publisher.
